# Competition and quality indicators in the health care sector: empirical evidence from the Dutch hospital sector

**DOI:** 10.1007/s10198-016-0862-6

**Published:** 2017-01-03

**Authors:** R. R. Croes, Y. J. F. M. Krabbe-Alkemade, M. C. Mikkers

**Affiliations:** 1 NZa (Dutch Healthcare Authority) and Erasmus University Rotterdam, Rotterdam, The Netherlands; 2 NZa (Dutch Healthcare Authority) and Free University Amsterdam, Amsterdam, The Netherlands; 3 NZa (Dutch Healthcare Authority) and Tilburg University, Tilburg, The Netherlands

**Keywords:** Competition, Quality, Hospitals, Market structure, I 110, L 110, L 130

## Abstract

There is much debate about the effect of competition in healthcare and especially the effect of competition on the quality of healthcare, although empirical evidence on this subject is mixed. The Netherlands provides an interesting case in this debate. The Dutch system could be characterized as a system involving managed competition and mandatory healthcare insurance. Information about the quality of care provided by hospitals has been publicly available since 2008. In this paper, we evaluate the relationship between quality scores for three diagnosis groups and the market power indicators of hospitals. We estimate the impact of competition on quality in an environment of liberalized pricing. For this research, we used unique price and production data relating to three diagnosis groups (cataract, adenoid and tonsils, bladder tumor) produced by Dutch hospitals in the period 2008–2011. We also used the quality indicators relating to these diagnosis groups. We reveal a negative relationship between market share and quality score for two of the three diagnosis groups studied, meaning that hospitals in competitive markets have better quality scores than those in concentrated markets. We therefore conclude that more competition is associated with higher quality scores.

## Introduction

Many countries are facing high healthcare costs, which are also continuing to rise steadily. As a response to these trends, reforms have been implemented with the aim of controlling healthcare costs and improving the quality of care, and it seems likely that further such reforms will be put in place. The Netherlands is an example of a country that has recently reformed its healthcare system. The reforms took place in 2006 and included essential competitive elements [28].

The main objectives of the reforms to the healthcare system were to reduce costs, increase the quality and accessibility of healthcare and, at the same time, to maintain an equitable healthcare system. Today, the Dutch system can be characterized as a system of managed competition in which health insurers compete for subscribers and healthcare providers compete for contracts with health insurers. A prospective payment system has been implemented to support negotiations between health insurers and hospitals. The prices of the hospital products, called Diagnosis Treatment Combinations, are in part set by the government. Prices for complex and relatively low-volume care are regulated (representing around 30% of hospital production in 2012). Other prices (mainly for elective care) have been liberalized and are set after negotiations between insurers and hospitals [3, 29].

Any healthcare system requires quality information regarding the care provided [33]. For this, an adequate system of outcome and quality measurements is necessary and information based on the insights provided by this system must be available to consumers. In the Netherlands, quality information became publicly available in 2008 via the website http://www.KiesBeter.nl. Since then, consumers have been able to compare the quality of treatment for specific medical conditions at all Dutch hospitals [32]. Consumers are obliged to take out basic health insurance covering all basic healthcare including hospital care.

In this paper, we investigate the relationship between competition and quality indicators for hospital products in a market in which prices are negotiated between health insurers and hospitals. We contribute to the literature in several ways. We contribute to our knowledge of the relationship between competition and quality. The majority of the existing literature on this subject focuses on the US and UK healthcare systems (for example [5, 6, 14, 21, 23, 24, 26]. We, however, examine a European country with mandatory health insurance. Secondly, we assess the impact of competition on quality in an environment of liberalized prices. For this research, we used unique price and production data relating to three diagnosis groups (cataract, adenoid and tonsils, bladder tumor) produced by Dutch hospitals and Independent Treatment Centers in the period 2008 to 2011. Some 928,544 claims with a total revenue of 1.3 billion euros were examined. Thirdly, most other papers do not model competition.^1^ We use a model to measure market power in a framework that is rooted in economic theory [12]. Fourthly, we have also taken into account the Independent Treatment Centers (ITCs), which have entered the market in recent years, when measuring market power; many studies do not take the rising number of ITCs into account [7].

A total of 178 ITCs were licensed to provide health care services in the Dutch hospital market. Particularly in the field of ophthalmology, it is important to include ITCs in this research because ITCs are responsible for more than 10% of the market for cataract treatments [22].^2^ The results reveal a negative relationship between market share and quality scores, meaning that hospitals in competitive markets achieve higher scores on quality than those in concentrated markets. The paper will proceed as follows. In “Institutional context” we will give a brief introduction of the institutional context of the healthcare system in the Netherlands. In “Literature”, we will provide an overview of the relevant theoretical and empirical literature. We will then proceed to describe our estimation strategy and data in “Estimation strategy”, while the results are given in “Estimation results” and “Further examination of the empirical model”. “Conclusion and discussion” will outline our conclusions.

## Institutional context

In 2006, the healthcare system in the Netherlands underwent extensive reform when managed competition was introduced.^3^ The system is based on two fundamental pillars: competition and solidarity. The basic concept is that insurers compete for policy holders and healthcare providers compete for contracts with insurers. The idea is that health insurers contract with individual health professionals and healthcare institutions, and negotiate terms relating to service delivery, price, quality, and volume of the health care production [27]. The selective contracting of health care providers is permitted. In order to support the negotiations between hospitals and insurers, a prospective payment system was put in place. Quality information for different hospital treatments was also made publicly available.

To guarantee both income solidarity and risk solidarity, the government introduced a mandatory health insurance scheme for the entire population in 2006. The Health Insurance Act (Zorgverzekeringswet 2005) includes several requirements that safeguard equal access to healthcare for everyone. The act obliges citizens to buy a basic benefits package from a private insurance company and obliges insurers to accept clients without premium differentiation. This basic benefits package is legally defined and includes hospital care, general practitioner care, dental care for children under 18 years, obstetrician care, maternity home care, ambulance services, and curative mental healthcare. The premium for this basic package is roughly 50% of the expected health care cost per capita. The other 50% is paid to the government as an income-dependent premium. The government pays the sum of the income-dependent premiums into a risk adjustment fund. This fund redistributes the money via risk-adjusted payments to the insurers. The system involves virtually no co-payments: the government currently prescribes a mandatory deductible of €375 and an optional deductible (between €0 and €500). In the period that our research relates to (2008–2011), the mandatory deductible increased from €150 to €170, and the optional deductible was between €0 and €500.^4^

## Literature

### Theoretical model

Economic theory on quality levels is highly equivocal and quality levels seem to be highly dependent on the market structures that are in place. Hospitals providing comparable health services may vary in the level of price-quality provided. It is important to identify the factors that drive this relationship. In price-regulated markets, quality is the only dimension on which one can compete. In this setting, quality levels depend on whether price-cost margins are positive or negative [12]. In non-price-regulated markets, where providers are able to determine price and quality level, quality levels depend on elasticities of demand for price and quality. If quality information is not transparent, competition will focus on the price dimension [24].

For our setting, which involves managed competition, Gaynor and Town [12] provide a relevant theoretical model. Given that in the Netherlands hospitals and insurers bargain with each other, it is most useful to analyze competition and quality through a bargaining framework. Gaynor and Town [12] (pages 566–568) present a model by which hospital and insurers bargain on price and hospitals are allowed to determine quality levels. In this framework, an insurer constructs a network of hospitals by bargaining with hospitals for their inclusion in that network. Insurers sell this network of hospitals to consumers through a health plan. The desirability of a network to a consumer depends the value that he/she attaches to the hospitals that are included in that network in the event that he or she needs treatment.

The bargaining model of Gaynor and Town [12] consists of four phases.

1. Each hospital determines its quality level
2. Insurers and hospitals negotiate prices (if they agree, the hospital can become part of the health plan network)
3. Patients choose a health plan based on their preferences
4. In the event that they need treatment, patients choose a hospital from the network

Gaynor and Town [12] find that the impact of competition on quality is generally ambiguous. However, in the event that hospital demand is not responsive to price—which is actually the case in our setting, see “Institutional context”—then the impact of competition on quality is positive. Any increase in competition induces all hospitals to increase their quality. On balance, the effect of this increase in competition on hospitals’ prices depends on their relative bargaining position after their increase in quality. If their relative bargaining position is unchanged, then there will be no price effect. However, if there is a change in their relative bargaining position, due to, for example, differences in their marginal costs for achieving quality standards, then hospital with lower marginal costs for achieving quality will choose a relatively higher quality and improve their relative bargaining positions and, therefore, their prices. Using this framework as a guide for our empirical model, we would conjecture that in the Dutch market, we can expect a positive relationship between quality and competition.

### Empirical literature

Various studies have examined the relationship between competition and quality in relation to price negotiations between hospitals and health insurers. In their literature review, Gaynor and Town [12] conclude that the impact of competition on quality remains ambiguous, ranging from negative to positive. Most studies undertaken originate in the US or UK. Lyon [19] examined the relationship between competition and quality for the managed care market. Lyon [19] showed that when there is greater price competition, price and quality are both lower than in traditional markets. Lyon’s [19] model shows that competition has a positive impact on quality when patients have a free choice of hospitals. When patients hospital choice is restricted, competition may lead to excessively low or high quality. Also, Gowrisankaran and Town [14] estimate the effect of competition on quality by comparing Medicare patients and HMO patients. Their assumption is that hospitals provide the same quality for all patients and that a change in competition will influence changes in quality for the hospital as a whole. Hospitals cannot influence Medicare prices because the government sets prices. For Medicare patients, the level of reimbursement determines whether hospitals will adjust quality (when the price is lower than cost, hospitals are incentivized to reduce quality and vice versa). For two diagnoses, pneumonia and acute myocardial infarction, these authors use mortality rates as quality measure. The results show that for HMO patients, competition leads to lower hospital prices and higher hospital quality. For Medicare patients, their results show that competition leads to lower quality, indicating that Medicare margins are (excessively) small. Encinosa and Didem [8] estimated logit regressions to examine the relationship between safety outcomes and hospital profit margins and find a gradual negative relationship, meaning that pressure on hospital finance leads to lower quality. Escarce et al. [9] examine the relationship between competition and quality in three US states with different levels of HMO penetration. Their logistic regression models reveal a positive relationship in states with the highest average market competition measures and HMO penetration.

Propper et al. [24] examine the effect of price competition on quality with a difference-in-difference model in the UK. In this model, competing hospitals are compared with non-competing hospitals based on geographic location. They use AMI mortality as an unobserved quality measure. This study finds a negative relationship between competition and AMI death rate. However, the relationship between an observed quality measure (the waiting list for elective care procedures) and competition is positive.

An important contribution outside the hospital market is Forder and Allan [10], who study the impact of competition on quality and price of English care and nursing homes. They show that competition reduces both quality and prices in nursing homes. A major difference in the institutional setting, however, is that a considerable number of the residents in these nursing homes are ‘self-payers’, which makes consumers much more price-sensitive than the consumers in studies on the hospital market.

In the Netherlands, two papers have examined the competition-quality relationship [2, 15]. Bijlsma et al. [2] used outcome and process-quality indicators after managed competition was introduced in Dutch hospitals. They used the Basic Data Set of the Health Inspectorate from the period 2004–2008. Most of these indicators were at the hospital level rather than at the diagnosis level. Competition is based on fixed radius measures. Although the relationship between competition and some process indicators was positive and significant, they do not find a quality-competition relationship in the quality of outcomes. Heijink et al. [15] studied the relationship between price, volume, and quality for elective cataract surgery in the Netherlands and found little variation in cataract quality indicators between Dutch hospitals after the introduction of price competition [15]. Other Dutch studies analyzed the relationship between patient hospital choice and quality with publicly available quality data. Both studies found significant patient sensitivity to quality data [33, 1]. Varkevisser et al. [33] examined this relationship specifically in relation to angioplasty treatments, which is a treatment for which hospitals require government permission and which is price-regulated. Even when quality information is noisy (for example because quality data is not adjusted for case mix), this relationship holds. Beukers et al. [2] studied this relationship with regard to hip replacements in the period 2008–2010. Their logit regressions indicate that although the relationship between quality and patient hospital choice is significant, travel time is a more important indicator of patients choice of hospital.

## Estimation strategy

### Empirical model

To determine the relation between quality and competition, we estimate a panel data model. We consider the following general linear model:

1 $$\begin{aligned} y_{it}= \varvec{x}_{it} \varvec{\beta }+v_{it}, \end{aligned}$$

where *t* denotes the year $(t=1,2,\ldots ,T)$ and *i* denotes the hospital ($i=1,2,\ldots ,N$). The independent variables for hospital *i* in year *t* are given by vector $\varvec{x}_{it}$ and the dependent variable is given by $y_{it}$. In this model $v_{it}=u_{it}+c_{i}$ is the composite error, where $c_{i}$ is the unobserved component and $u_{it}$ is the idiosyncratic error (see for example [34]). For each model, we made assumptions on the correlation between the unobserved component $c_{i}$ and $\varvec{x}_{it}$. If we allow these to be correlated, then we have a fixed-effects model where $c_{i}$ is a parameter that we will estimate. If we assume that they are uncorrelated then we have a random-effects model in which we assume a structure for the variance of $v_{it}$ (see [34]).

In our specific application, we estimated a model for each diagnosis. For hospital *i* in year *t* $(t=2008,\ldots ,2011)$ we denoted its concentration index by *ms* (see below for the definition) and its quality by *quality*. To control for possible case mix differences between hospitals, we included the fraction of females ($\mathrm{frac}\_\mathrm{female}_{it}$) and the fraction of patients who are 65 years old or older ($\mathrm{frac}\_65_{it}$). To control for the fact that some hospitals deal with one dominant health insurer, while other hospitals deal with several competing insurers, we calculated the HHI of the insurers that a hospital faces ($\mathrm{HHI}\_\mathrm{ins}_{it}$). We calculated this for, say, hospital *i* in year *t*, by summing up the squared shares that each insurer has in the total revenue of hospital *i* in year *t*. Furthermore, given that teaching hospitals may treat more severe patients, we included a dummy variable for teaching hospitals ($\mathrm{acad}_{it}$) and since quality may depend on volume, we included a dummy for hospitals with a low volume of patients ($\mathrm{lowvolume}_{it}$). In each year, the $25\%$ of hospitals with the lowest volume were considered as low-volume hospitals.

The estimated model is:

2 $$\begin{aligned} {\text{quality}}_{{it}} & = \beta _{1} ms_{{it}} + \beta _{2} {\text{frac}}\_{\text{female}}_{{it}} + \beta _{3} {\text{frac}}\_65_{{it}} \\ & + \beta _{4} {\text{HHI}}\_{\text{ins}}_{{it}} + \beta _{5} {\text{lowvolume}}_{{it}} + \beta _{6} {\text{acad}}_{{it}} + v_{{it}} \\ \end{aligned}$$

To estimate the relationship between quality and competition, we needed to measure market power. There has been a great deal of debate about market definition and the measurement of market power in the literature. For an overview, see [12]. Many authors use a rather crude measure of competition: for example [24], measure competition as number of hospitals within a 30-min journey controlled for population density. However, although travel time is an important factor in hospital choice, choices can also be influenced by other patient and hospital characteristics.

Gaynor and Vogt [12] propose the use of the Logit Competition index (LOCI) to measure market power in the hospital market. The index is based on a weighted average of a hospitals market share per micro-market. The construction of the competition index starts by modeling the demand with a choice model. The choice model includes a utility function which, given characteristics of the consumer and hospital, depends on the utility that a patient derives from each hospital. The utility depends on both observable and non-observable consumer and hospital characteristics. With the logit choice model, it is possible to calculate the probability that a specific consumer type will choose a specific hospital. Each group of patients with similar characteristics (e.g., zip-code, age, gender, diagnosis, etc.) forms a micro market.

Under a standard price competition model, the competition index (LOCI) of hospital *j* for consumer type *t* is given by (see [12])

$$\begin{aligned} \Lambda _{j}= \displaystyle \sum _t w_{tj}(1-s_{tj}) \end{aligned}$$

where the weights $w_{tj}$ are the relative importance of each consumer type

$$\begin{aligned} w_{tj}= \frac{N_{t} s_{tj}}{\displaystyle \sum _t N_{t} s_{tj}} \end{aligned}$$

and $N_t$ is the number of consumers of the type *t*.

The LOCI $\Lambda _{j}$ is a measure of the competitiveness in the market. The index takes on values between 0 and 1, where $\Lambda = 0$ means that hospital *j* is monopolist and $\Lambda = 1$ means that the market is perfectly competitive.

We interpret the LOCI as 1 minus the weighted market share. To simplify the interpretation of our results with we use the variable *ms* as a shorthand for “market share”, which is constructed as $(1-\Lambda )$.

For our purposes, we are able to use actual market share with the advantage that all non-observable characteristics are implicitly taken into account. Alternatively, we could have used estimated market share, with the advantage that all consumers are taken into account. In our estimations, we used actual market share. However, the use of actual market share could potentially lead to endogeneity: hospitals providing good quality may have higher market share.

Our approach is similar to other articles about the impact of hospital competition on the quality of healthcare, e.g., Gowrisankaran and Town [13, 14], Kessler and McClellan [16], Gaynor et al. [11], and Cooper et al. [6] all use a market share based on travel distance in their estimations directly or in their IV estimations, in order to avoid potential endogeneity problems. Our approach is different from Forder and Allan [10]: they use an administrative region (Medium-level Super Output Area) as a market to calculate market share. Since they do not use patient choice models, they cannot rely on market share based on travel distance as IV. They therefore use the level of competition in neighboring areas as IV.

To prevent any endogeneity problems, we also estimate our regressions with an estimated market share (based on travel distance only) in an instrumental variable (IV) approach. Our main contribution is that we define our micro markets at the level of the quality indicator. Our micro markets consisted of the group of DTCs that are linked to the quality indicators. For each quality indicator, we estimated the relationship between the indicator and the competition indicator, which meant that we were able to construct a competition indicator for each quality indicator. The micro markets are defined by a four-digit zip code and diagnosis. The narrower a micro market becomes, the more precise the total market share becomes. However, we should not make our micro markets smaller than four-digit zip codes and diagnosis, because then we would have too few observations per zip code. For example, age is highly skewed for each diagnosis: the cataract and bladder tumor diagnosis groups include mainly elderly patients, while the tonsil diagnosis group consists mainly of younger people. This indicates that splitting the micro markets across age categories will not add a great deal of information. Furthermore, there is no reason to assume that choices would depend on gender.

### Quality indicators

For the purposes of this research, we used the quality indicators from the ‘Dutch Healthcare Transparency Program’ (in Dutch: Zichtbare Zorg), which were developed by the Health Inspectorate in order to support various goals such as the provision of information for patients and consumers to help them make their choices, purchase information for health insurers, control information for the Inspectorate and improvement information for providers. The Dutch Healthcare Transparency Program started in 2007 and in 2008 quality indicators became available for ten diagnosis groups. The hospitals are required to provide the registered quality indicators to the government annually.^5^ Independent Treatment Centers are not obliged to provide quality indicators, and, for this reason, we have no data on the quality indicators of the Independent Treatment Centers. The quality indicators can be divided into process indicators, structure indicators, and outcome indicators.

Structure indicators relate to the organization and are recorded at the hospital level; process indicators measure the process of activities at the patient level and outcome indicators measure outcome values at the patient level. Although outcome indicators are the most important indicators in terms of informational value, the share of outcome indicators for the Dutch Healthcare Transparency Program is less than 16%. According to an evaluation carried out by the Court of Audit (in Dutch: Algemene Rekenkamer), the indicators of the Dutch Healthcare Transparency Program indicators for the hospital sector are stable and it is therefore possible to analyze trends over the years for which records have been kept [25].

We used quality indicator data from 2008–2011. Because our time period is relatively short, we used process and structural indicators (with a ratio scale) rather than outcome indicators because process and structure indicators can be influenced by hospital management and not only by medical specialists, and these types of indicators are also used by insurers [4]. Six selection criteria were applied to include quality indicators for the diagnosis groups in our research sample. (1) The diagnoses are not selected on medical similarity but on whether hospitals can compete for patients. We used quality indicators for the diagnosis groups that made up the market segment. In this segment, the prices for the diagnosis groups are determined by negotiations between insurers and hospitals. This means that hospitals are able to compete on price and quality. This is not the case for all hospital treatments (such as urgent care). The Dutch Healthcare Authority has selected diagnoses for the market segment on criteria such as the transparency of the product definition, price and quality, the existence of market dynamics including entry and exit, the absence of undesirable effects and the absence of high transaction costs [17]. (2) Hospitals have been obliged to record quality indicators for the diagnosis groups since 2008; however, the number of diagnosis groups has grown over the years. We selected only the quality indicators that have been recorded since 2008. This means that the quality indicators that have been developed by the Health Inspectorate since 2008 are not part of our selection. (3) The quality indicators needed to be comparable over the years. (4) We selected diagnosis groups that involved surgical intervention. (5) We selected high-volume diagnosis groups with over 10,000 treatments per year. (6) We excluded indicators with categorical values (yes or no answer). Our final sample consisted of quality indicators for three diagnosis groups: cataract (ophthalmology), adenoid and tonsils (otolaryngology), and bladder tumor (urology). For bladder tumor, quality indicator data were available for 2008 to 2010 and for cataract, and data were available for 2008 to 2011 for adenoid and tonsils. The three diagnoses are elective care treatments that include daycare surgery. It should be noted that this is also the case for the bladder tumor diagnosis because the quality indicator that was used in this study relates to the low-risk patient group (non-muscle invasive bladder tumor). Table 1 shows the quality indicators that we included in our analysis.^6^

**Table 1:** Quality indicators

Indicator code	Description	Type	Average	SD
Cataract				
i02-02	Complications: percentage of cataract operations *without* a posterior capsular rupture (including vitrectomy)	Process	99.63	0.35
i02-03a	Accurate diagnosis of the second eye: percentage of patients undergoing a cataract operation on both eyes with a gap of more than 28 days between the first and second operation. For a careful assessment of the second eye there should be enough time between the surgery of the first and second eye	Process	95.56	8.40
i02-03b	Accurate diagnosis of the second eye: percentage of patients undergoing a cataract operation on both eyes that have (*i*) a postoperative check of the first surgery before the second operation and (*ii*) a gap of more than 14 days between the first operation and the last postoperative check for the first operation	Process	87.63	23.21
Bladder tumor				
i01-02	Vesicoclysis: percentage of non-muscle invasive bladder cancer patients with a trans-urethral resection of the tumor (TURT) that have a washing out of the urinary bladder within 24 h after the TURT	Structure	69.98	23.66
Adenoid and tonsils				
i10-02	Preoperative consultation: percentage of tonsillectomy patients screened at an anesthesiology outpatient clinic before tonsillectomy	Process	90.80	23.58
i10-04a	Postoperative pain measurement: percentage of inpatient tonsillectomy patients that have their pain intensity measured every 8 h	Process	84.76	21.61
i10-04b	Postoperative pain measurement: percentage of measured inpatient patients *without* serious pain (i.e., Visual Analog Scale for Pain $\le$ 7 or Numeric Rating Scale $\le$ 7)	Process	93.32	14.51
i10-04c	Postoperative pain measurement: percentage of daycare patients that have been telephoned after their operation to monitor their pain intensity	Process	75.47	38.63

This table contains for each quality indicator its average score and standard deviation pooled over the years

The indicators measure various aspects of the quality of care. For example, for cataract i02-02 measure the complication rate, while i02-03b measure the diagnosis process. We can indeed observe that (i) i02-02 have a higher average score than i02-03b and (ii) i02-02 have a lower standard deviation than i02-03b. This is unsurprising, since a one percentage-point change in i02-02 has more direct clinical relevance than a one percentage-point change in i02-03b. For this reason, it is more useful to compare changes in terms of one standard deviation. Note that i02-02 is an outlier with respect to the standard deviation. The other indicators have more similar standard deviations and clinical relevance.

In order to compare the quality indicators over the years and with one another, we rescaled and aggregated the indicators as follows: for each year *t* $(t=1,2,\ldots ,n)$ and diagnosis group *k* ($k=1,2,\ldots ,K$) we calculated a combined quality indicator score per hospital *i* ($i=1,2,\ldots ,N$). First, each indicator *h* $(h=1,2,\ldots ,H)$ of, say, diagnosis group *k* is rescaled to a *z*-score (*z*-score of indicators have an average value of zero and a standard deviation of 1):

$$\begin{aligned} z_{i{th}}= \frac{(P_{i_{th}}- \mu _{ th})}{sd_{ th}} \end{aligned}$$

where $z_{ith}$ is the value of the indicator *h* of diagnosis group *k* for hospital *i* in year *t*, $\mu_{th}$ is the average value of indicator *h* in year *t* and $sd_{th}$ is the standard deviation of indicator *h* in year *t*. Note that for all indicators that a high *z*-score is associated with high quality of care and low *z*-score is associated with low quality of care.

Secondly, we calculate for each hospital in each year we calculated an average *z*-scores for diagnosis group *k*, which we denote by $\mathrm{quality}_{itk}$, by averaging over $z_{ith}$:

$$\begin{aligned} \mathrm{quality}_{itk}= \frac{\sum _{h=1}^{H} z_{ith}}{H}. \end{aligned}$$

We thus interpreted $\mathrm{quality}_{itk}$ as the diagnosis group *k* (combined) quality indicator of hospital *i* in year *t*.

### Data

This research was based on claims data from 2008 to 2011 from all Dutch hospitals and Independent Treatment Centers (ITCs). The Dutch hospital market consists of 87 hospitals, two specialized hospitals, and eight academic hospitals. The total number of ITCs rose from 189 in 2008 to 282 in 2012. Our unique dataset consists of patient-level data including patient characteristics such as gender, age, zip code, diagnosis and treatment, hospital characteristics and hospital contract prices for our three selected diagnoses. The total number of patients in the period 2008 and 2011 for cataract was 474,410 with a total revenue of 843 million euros. The total number of patients in the period 2008 and 2011 for adenoid and tonsils was 223,177 with a total revenue of 219 million euros. The total number of patients in the period 2008 and 2011 for bladder tumor was 46,497 with a total revenue of 244 million euros.

The calculation of the weighted market share was based on the claims data from the hospitals and Independent Treatment Centers (ITCs), where we removed those cases with invalid zip codes (which amounted to less than 1% of our sample). Thus, when calculating the market share, we were able to take into account the (potential for) competitive pressure from ITCs. However, as discussed in “Quality indicators”, we had no data on the quality indicators for the ITCs. This implies that the analysis of the quality indicators is restricted to hospitals and excludes the ITCs. For each diagnosis, we removed those hospitals where there was no data on quality indicators. Tables 2, 3, and 4 present the descriptive statistics for the variables used in our empirical model at the diagnosis level. The total number of observations ranges from 191 for tonsils to 286 for cataract. For example, the average actual market share *ms* for cataract was 0.58 (SD 0.21) with a minimum of 0.06 and maximum of 0.97 meaning that there are large differences between the market share of hospitals. The other diagnoses display comparable actual market share. The average hospital-insurer HHI is moderately strong and ranges from 0.39 for cataract, 0.32 for tonsils, and 0.38 for bladder tumor.

As mentioned in “Quality indicators”, we have used standardized quality scores for each diagnosis. The standardized quality scores ranged between $-3.05$ and 0.78 for cataract, between $-2.17$ and 0.71 for tonsils and between $-2.85$ and 1.42 for bladder tumor.

We included two hospital characteristics in our empirical model. Firstly, we used a dummy variable to control for whether a hospital is a general hospital or academic (university) hospital, a low-volume dummy, and insurance-hospital HHI. For each diagnosis, less than 10% of the hospitals included were academic hospitals. To control for patient characteristics, we included three variables: the fraction of female patients, the fraction of patients that were older than 65 years, and the co-morbidity index. The results differed per diagnosis due to the characteristics of the condition and the set of hospitals included in the analysis. The co-morbidity index, which is defined in this study as the average number of diagnoses, varied considerably between the diagnoses. The co-morbidity for tonsils had the lowest index of 1.04. The average number of additional diagnoses for bladder tumor (3.19) and cataracts (2.18) was higher due to the older population involved.

**Table 2:** Cataract

Statistic	*N*	Mean	SD	Min	Max
*ms*	286	0.58	0.21	0.06	0.97
Quality	286	0.00	0.63	−3.05	0.78
frac_female	286	0.59	0.03	0.51	0.68
frac_65	286	0.83	0.06	0.58	0.91
Com	286	2.18	0.30	1.60	3.17
HHI_ins	286	0.39	0.11	0.19	0.63
Lowvolume	286	0.25	0.43	0	1
Acad	286	0.09	0.28	0	1

This table shows summary statistics for each diagnosis group at the hospital-year level (2008–2011). We report the average, standard deviation, minimum, and maximum of the variables that we included in our regression analysis. We also show the total number of observations for each diagnosis group

**Table 3:** Adenoid and tonsils

Statistic	*N*	Mean	SD	Min	Max
*ms*	191	0.69	0.19	0.12	0.98
Quality	191	0.00	0.59	−2.17	0.71
frac_female	191	0.51	0.03	0.42	0.60
frac_65	191	0.003	0.003	0.00	0.02
Com	191	1.04	0.30	0.53	2.18
HHI_ins	191	0.32	0.09	0.17	0.57
Lowvolume	191	0.25	0.43	0	1
Acad	191	0.07	0.26	0	1

This table shows summary statistics for each diagnosis group at the hospital-year level (2008–2011). We report the average, standard deviation, minimum and maximum of the variables that we included in our regression analysis. We also show the total number of observations for each diagnosis group

**Table 4:** Bladder tumor

Statistic	*N*	Mean	SD	Min	Max
*ms*	199	0.73	0.15	0.33	0.98
Quality	199	0.00	1.00	−2.85	1.42
frac_female	199	0.22	0.05	0.10	0.45
frac_65	199	0.72	0.06	0.52	0.86
Com	199	2.68	0.46	1.70	4.32
HHI_ins	199	0.38	0.11	0.19	0.71
Lowvolume	199	0.23	0.42	0	1
Acad	199	0.10	0.29	0	1

This table shows summary statistics for each diagnosis group at the hospital-year level (2008–2010). We report the average, standard deviation, minimum and maximum of the variables that we included in our regression analysis. We also show the total number of observations for each diagnosis group

## Estimation results

Tables 5, 6, and 7 present the estimation results for each diagnosis group using the pooled OLS estimator, fixed effect estimator, and random-effects estimator. For each estimation method, we present two models: a model with and a model without control variables. Because the estimated standard errors could be heteroskedastic, we used the Mackinnon and White [20] Heteroskedasticity-Consistent standard errors.

We performed the Hausman test to determine whether we should select the fixed or random effect model: given that (i) the fixed effect model is consistent if the unobserved component and observable variables are correlated and (ii) the random effect model is inconsistent, the presence of significant differences in the estimated coefficients indicates that there is correlation between the unobserved component and observable variables and, thus, that we should discard the random-effects model [34]. For all models, the Hausman test accepts the null hypotheses that there is no difference between the coefficient of the random and fixed-effects model (for each model, the *p* value is larger than 0.10). This indicates that the random-effects models are consistent and, therefore that we can use the results of the random effect models to determine the relationship between quality indicators and market share.

**Table 5:** Regression results bladder tumor

	Dependent variable					
	Quality					
	Pooled	Pooled	Fixed effects	Fixed effects	Random effects	Random effects
	(1)	(2)	(3)	(4)	(5)	(6)
Constant	1.275***	2.058			1.348***	2.803**
	(0.338)	(1.347)			(0.360)	(1.265)
*ms*	−1.731***	−1.764**	−2.504*	−3.331**	−1.841***	−2.241***
	(0.492)	(0.705)	(1.381)	(1.695)	(0.510)	(0.674)
frac_female		−1.136		0.066		−0.149
		(1.483)		(1.262)		(1.019)
frac_65		−0.173		−1.975		−1.001
		(1.686)		(1.765)		(1.425)
Com		0.018		−0.241		−0.106
		(0.205)		(0.159)		(0.158)
HHI_ins		−1.019		1.469		−0.134
		(0.889)		(1.217)		(0.801)
Lowvolume		−0.165		−0.004		−0.200
		(0.260)		(0.231)		(0.188)
Acad		−0.126				−0.286
		(0.271)				(0.300)
Observations	199	199	199	199	199	199
$R^{2}$	0.069	0.088	0.029	0.073	0.050	0.063
Adjusted $R^{2}$	0.068	0.084	0.017	0.040	0.049	0.060

* $p<0.1$; ** $p<0.05$; *** $p<0.01$. We report the results from the pooled, fixed-effects, and random-effects model. For each model, we report two variants: (i) a simple model with the weighted market share regressed on the average scaled quality indicator and (ii) a model with additional control variables. We report the MacKinnon and White Heteroskedasticity-Consistent standard errors (in parentheses under coefficients). We used data from 2008 to 2010

**Table 6:** Regression results cataract

	Dependent variable					
	Quality					
	Pooled	Pooled	Fixed effects	Fixed effects	Random effects	Random effects
	(1)	(2)	(3)	(4)	(5)	(6)
Constant	0.122	−2.552*			0.149	−2.631**
	(0.145)	(1.550)			(0.149)	(1.237)
*ms*	−0.199	−0.584**	−1.481	−1.923**	−0.264	−0.772**
	(0.259)	(0.292)	(1.023)	(0.883)	(0.261)	(0.325)
frac_female		2.541		1.208		1.718
		(1.925)		(2.168)		(1.830)
frac_65		1.398		1.354		1.732
		(1.294)		(1.703)		(1.234)
Com		0.072		0.479		0.216
		(0.228)		(0.377)		(0.235)
HHI_ins		0.196		3.568*		0.467
		(0.680)		(2.082)		(0.691)
Lowvolume		0.069		−0.108		−0.017
		(0.120)		(0.154)		(0.127)
Acad		−0.196				−0.229
		(0.313)				(0.287)
Observations	286	286	286	286	286	286
$R^{2}$	0.004	0.072	0.014	0.070	0.004	0.051
Adjusted $R^{2}$	0.004	0.070	0.010	0.048	0.004	0.050

* $p<0.1$; ** $p<0.05$; *** $p<0.01$. We report the results from the pooled, fixed-effects, and random-effects model. For each model, we report two variants: (*i*) a simple model with the weighted market share regressed on the average scaled quality indicator and (*ii*) a model with additional control variables. We report the MacKinnon and White Heteroskedasticity-Consistent standard errors (in parentheses under coefficients). We used data from 2008 to 2011

**Table 7:** Regression results adenoid and tonsils

	Dependent variable					
	Quality					
	Pooled	Pooled	Fixed effects	Fixed effects	Random effects	Random effects
	(1)	(2)	(3)	(4)	(5)	(6)
Constant	−0.008	0.292			−0.089	−0.064
	(0.236)	(0.902)			(0.283)	(0.804)
*ms*	0.011	−0.068	0.723	0.382	0.099	−0.034
	(0.323)	(0.562)	(1.537)	(1.639)	(0.383)	(0.514)
frac_female		−0.388		−0.191		−0.101
		(1.452)		(1.158)		(1.215)
frac_65		−19.167		4.575		−17.188
		(25.528)		(19.921)		(23.471)
Com		0.257		0.379		0.304
		(0.198)		(0.422)		(0.201)
HHI_ins		−0.579		1.394		−0.233
		(0.602)		(1.915)		(0.608)
Lowvolume		−0.185		0.036		−0.156
		(0.231)		(0.182)		(0.178)
Acad		−0.224				−0.228
		(0.311)				(0.314)
Observations	191	191	191	191	191	191
R$^{2}$	0.00001	0.066	0.002	0.015	0.002	0.038
Adjusted R$^{2}$	0.00001	0.063	0.001	0.008	0.002	0.036

* $p<0.1$; ** $p<0.05$; *** $p<0.01$. We report the results from the pooled, fixed-effects, and random-effects model. For each model, we report two variants: (i) a simple model with the weighted market share regressed on the average scaled quality indicator and (ii) a model with additional control variables. We report the MacKinnon and White Heteroskedasticity-Consistent standard errors (in parentheses under coefficients). We used data from the period 2008–2011

Table 5 presents the estimation results for bladder tumor using six different models. The adjusted *R*-squared of these models ranges between 0.017 for the fixed effect model and 0.084 for the pooled model. From all the models shown in Table 5, we can conclude that the weighted market share (1 minus LOCI) is significantly related with scaled quality score. The estimated coefficients are significant at a level of 1% for the random-effects models and the pooled effect model without control variables. The other models are significant at a level of 5% (fixed effects with control variables and pooled model with control variables) and the 10% level (fixed effect model without control variables). This indicates that hospitals in competitive markets have higher quality scores for bladder tumor, which supports the hypothesis that greater competition leads to higher quality scores. Models 2, 4, and 6 include six control variables which control for the patient characteristics of the hospital (fraction of female patients, fraction of patients older than 65 years and co-morbidity index) and hospital characteristics^7^ including health insurer-hospital HHI, the type of hospital and low-volume dummy.

Tables 6 and 7 show the results for the diagnosis groups cataracts and adenoid and tonsils. The results of the models for cataract are comparable to the results for bladder tumor and reveal a negative relationship between market concentration and quality scores. Contrary to what we expected, the models for the diagnosis group adenoid and tonsils do not show any significant estimations.

For the cataract and bladder tumor diagnosis groups, we can show how to interpret the magnitude of the estimated relationship between quality scores and market share through an example. Consider the estimated difference in the quality scores for a hospital with the lowest market share (i.e., a market share of 0.06 for cataract, see Table 2) and the hospital with the highest market share (i.e., market share 0.98 for bladder tumor, see Table 4). Using the results of the random-effects model for bladder tumor presented in Table 6, the market share difference of 0.92 translates into a shift in quality scores of −2.06 standard deviations from the mean (95% confidence-interval: $[-3.39,-0.73]$). Similarly, using the results of the random-effects model for bladder tumor presented in Table 5, the market share difference of 0.92 translates into a shift in quality scores of −0.71 standard deviations from the mean (95% confidence-interval: $[-1.35,-0.069]$).

### Robustness check

To check the robustness of our model with aggregated quality indicators, we also estimated the pooled, fixed-effects, and random-effects model for each individual quality indicator separately (not relevant for bladder tumor). Table 8 shows the estimated market-share coefficients for each quality indicator. For the sake of clarity, we only show the estimated market-share coefficients. For each diagnosis, the sign of the estimated market-share coefficients are generally consistent with the aggregated model: negative for cataract and mixed for adenoid and tonsils.

**Table 8:** Disaggregated regression results for market share (*ms*)

	Dependent variable		
	Quality		
	Pooled	Fixed effects	Random effects
	(2)	(4)	(6)
Cataract: i02-02 ms	0.040	−2.812	−0.173
Cataract: i02-03a ms	−1.024**	−0.327	−1.038**
Cataract: i02-03b ms	−0.775**	−2.707*	−0.906**
Adenoid and tonsil: i10-02 ms	0.361	−2.316	0.018
Adenoid and tonsil: i10-04a ms	0.901	−3.107	0.744
Adenoid and tonsil: i10-04b ms	−0.369	3.018	−0.334
Adenoid and tonsil: i10-04c ms	−1.150*	3.782	−0.556

* $p<0.1$; ** $p<0.05$; *** $p<0.01$. We report the estimated market-share coefficient from the pooled, fixed-effects, and random-effects models, which also include the control variables. The estimated coefficient of the control variables are available from the authors by request. We report the MacKinnon and White Heteroskedasticity-Consistent standard errors (in parentheses under coefficients). We used data from the period 2008–2011

## Further examination of the empirical model

In our empirical estimations, we control for differences in patient and hospital characteristics. However, there is a potential source of bias in the estimation of the relationship between weighted market share and quality indicators: Firstly, as discussed in “Empirical model”, hospitals with a high-quality indicators may attract more patients than hospitals with low-quality indicators. This means that our regression models may suffer from an endogeneity problem and therefore give a biased result.

A potential issue is that hospitals may change their supply in response to their own or their competitors quality indicators. However, there were no indications that hospitals significantly restructured their supply:

- no new hospitals or new hospital locations were opened during the period of our study.
- no hospitals or hospital locations were closed during the period of our study.
- virtually no hospital increased or decreased their volume (number of patients) significantly.^8^
- the presence and entry of independent treatment centers (ITC) occurred mainly in the cataract diagnosis group. There was no ITC in the bladder tumor diagnosis group and only four ITCs in the adenoid and tonsils diagnosis group.

Secondly, hospitals and insurers negotiate the prices of the products in the diagnosis groups that we examined in a liberalized setting. An insurer is likely to be interested in the quality–price ratio and not the quality or price in isolation. Furthermore, as we noted in “Literature”, there may be a relationship between competition and prices through quality. In this section, we will examine both these issues.

### Endogeneity

To test whether our (pooled) regression model suffers from the endogeneity problem, we used the Wu–Hausman test. In this test the result of an instrumental variable (IV) model is compared to the result if the non-IV model.

To estimate an IV model, we must find an instrument for market share that meets two requirements: (1) it should be strongly correlated to market share and (2) it should be uncorrelated to the error term. We took an approach that has been commonly used in the health economics literature to deal with the endogeneity problem, namely to deploy IV instruments based on predicted patient flows (see for example [6, 16], and [11]). We estimated a conditional logit model that was based only on the travel time between the patient and hospital. By excluding the fixed-effects and other hospital and patient characteristics, we ensured that our predicted patient flows were exogenous to all patient preferences other then travel time. This implies that our patient flows were not influenced by the potential preferences that patients may have a hospital with a certain level of quality indicators.

In the conditional logit model, we estimated the utility that patient *t* would receive when he or she chose hospital *j* $(j,\ldots ,n)$, which we denote by $U_{tj}$. In our case, where we only use travel time as a predictor, the conditional logit model is given by:

$$\begin{aligned} U_{tj} = \alpha \mathrm{traveltime}_{tj} +\epsilon _{tj}, \end{aligned}$$

where the residuals $\epsilon$ are i.i.d. extreme value (see for example [31]). We used the same data as in “Estimation strategy”, with the addition of the travel times between the patient’s home and the hospital. The travel time was calculated as the driving time between the patient’s home and the hospital zip code. We estimated this model for each diagnosis group and year (see Table 9 for the estimation results). For cataract treatment, we took a random sample of the data for computability. In each year, the sample size was $50\%$ of the total patient population. As expected, we found that patients dislike traveling (negative coefficient for time travel).

**Table 9:** Result: conditional logit model

	Dependent variable		
	Hospital choice		
	Bladder cancer	Cataract	Adenoid and tonsil
	(1)	(2)	(3)
2008 traveltime	−0.220***	−0.232***	−0.195***
	(0.002)	(0.001)	(0.001)
2009 traveltime	−0.221***	−0.235***	−0.183***
	(0.002)	(0.001)	(0.001)
2010 traveltime	−0.231***	−0.236***	−0.179***
	(0.002)	(0.001)	(0.001)
2011 traveltime	−0.221***	−0.230***	−0.182***
	(0.002)	(0.001)	(0.001)
Number of patients 2008	9436	53,449	38,621
Number of patients 2009	9854	56,412	38,211
Number of patients 2010	10,122	57,391	39,032
Number of patients 2011	10,319	53,144	40,432

* $p<0.1$; ** $p<0.05$; *** $p<0.01$. For each year and diagnosis group, we report the results from the conditional logit model with travel time as the only predictor. For cataract we take a random sample. In each year, the sample size is $50\%$ of the total patient population

Given the fitted utilities $\hat{U}$’s from the estimated model, we can calculate for each patient the probability $\hat{P}_{ij}$ that this patient (*t*) chooses hospital *j*:

$$\begin{aligned} \hat{P}_{tj}=\frac{\hat{U}_{tj}}{\sum _k^n\hat{U}_{tk}}. \end{aligned}$$

We can now calculate the simulated weighted market share for each hospital in each year by replacing $s_{tj}$ with $P_{tj}$ in “Empirical model”. To determine the relevance of the instruments, we determined first-stage F-statistic, see for example [30]. For each model we found that the first-stage F-statistic was higher than 56 (*p* < 0.001). This indicates that our instruments were not weak.^9^ Subsequently, we re-estimated the pooled regressions in “Estimation results” by employing the simulated weighted market share as the instrumental variable (see Table 10 for the results). Given the IV-estimated coefficients, we could now carry out the Wu-Hausman test, in which we tested the hypothesis that there was significant difference between the coefficients from the pooled model and the coefficients from the IV model. For each diagnosis group we accepted the hypothesis that there was no difference (*p* value >0.90). We performed an additional IV estimation of the random-effects model (which is preferred to the fixed-effects model, see above). For bladder tumor, the estimated market-share coefficient is −2.45 (*p* = 0.062). For cataract, the estimated market-share coefficient is −0.94 (*p* = 0.107). For adenoid and tonsils, the estimated market-share coefficient is −0.55 (*p* = 0.35). These IV random-effects coefficient are very similar to the non-IV random-effects coefficient presented in the paper. The only difference is that the standard errors are larger, which is no surprise, since it is much more difficult to explain the differences than the levels of the quality indicators with an (IV) regression model. We were therefore able to conclude that our regression did not suffer from endogeneity bias.

**Table 10:** Result: pooled instrumental variable model

	Dependent variable		
	Quality		
	Bladder tumor	Cataract	Adenoid and tonsil
	(1)	(2)	(3)
Constant	2.094	−2.380	0.617
	(1.349)	(1.560)	(0.889)
*ms*	−1.829***	−0.753**	−0.603
	(0.707)	(0.294)	(0.545)
frac_female	−1.134	2.412	−0.317
	(1.485)	(1.922)	(1.433)
frac_65	−0.159	1.432	−18.521
	(1.691)	(1.311)	(26.590)
Com	0.016	0.046	0.193
	(0.205)	(0.229)	(0.202)
HHI_ins	−0.993	0.293	−0.285
	(0.889)	(0.681)	(0.612)
Lowvolume	−0.175	0.055	−0.235
	(0.261)	(0.120)	(0.229)
Acad	−0.137	−0.243	−0.384
	(0.271)	(0.325)	(0.290)
Observations	199	286	191
$R^{2}$	0.088	0.071	0.055
Adjusted $R^{2}$	0.084	0.069	0.053

* $p<0.1$; ** $p<0.05$; *** $p<0.01$. For each diagnosis group, we report the results from the pooled model, where we use the simulated weighted market share as an instrument for the weighted market share. The simulated weighted market share is based on a multinomial logit model. We report the MacKinnon and White (1985) Heteroskedasticity-Consistent standard errors (in parentheses under coefficients). We used data from 2008 to 2011

### Prices

As discussed above, there may be a relationship between prices and quality indicators. However, a comprehensive examination of the relationship between prices and quality indicators is beyond the scope of this paper. Instead, in this section we will provide a brief outline of this relationship. In “Literature”, we discussed the bargaining model by Gaynor and Town [12]. From this model, it follows that the effect of an increase in competition—through quality—on price depends on the effect that the increase in quality has on the relative bargaining position and therefore the prices charged by a hospital. We do not observe the factors that determine this effect such as the marginal cost of quality, see “Literature”. Generally, we would expect that hospitals and insurers to be interested in the quality-to-price ratio and not price or quality in isolation.

To provide an indication of the relationship between quality and prices, we estimated the same model as our quality indicator-market share model in “Empirical model”, where we included the relative prices as the independent variable rather than market share. We then estimated the pooled, fixed-effects, and random-effects models. In general, we found no significant results for the price variable.^10^ Our results indicate that, for the hospital products examined, having a relatively higher (or lower) quality indicators was not related to higher (or lower) prices during the period that we studied. We can therefore conclude that hospitals with better quality indicators are not compensated by higher prices.

## Conclusions and discussion

In this study, we have examined the impact of competition on the quality of healthcare for the Dutch hospital market. The Dutch government reformed the health care system in 2006 introducing managed competition in a context where income and risk solidarity are guaranteed. With this system, the government aims to reduce costs and increase quality of care. Health insurers compete for policy holders and healthcare providers compete for contracts with health insurers. We used a unique data set including individual patient-level claim data and information on quality indicators for three diagnosis groups—cataracts, adenoid, and tonsils and bladder tumor—produced by Dutch hospitals and Independent Treatment Centers.

For cataract and bladder tumor, the relationship between market share and quality scores was found to be significant. The robustness checks confirmed these results. The regression estimators for adenoid and tonsils were not significant. One possible explanation is that the patient group for adenoid and tonsils is less complex. It is mainly children younger than 11 years who are treated for adenoid and tonsils. This type of patient is less complicated and has fewer additional diagnoses compared to patients with, for example, bladder tumor (the fraction of patients older than 65 years is 0.72 for bladder tumor, 0.83 for cataract, and 0.01 for adenoid and tonsils, respectively).

Because of endogeneity, we could not include price as independent variable in our quality indicator models. To give an indication of this relationship, we replaced the market-share variable with the relative prices as the independent variable. We conclude from these models that there is no relationship between price and quality scores, which means that hospitals with higher-quality scores do not have higher prices.

Overall, we conclude that more competition leads to better published quality scores. This research does have some limitations however. For this research, we used observed quality information that did not include mortality rates (an outcome indicator). For our research period data, no data on mortality rates was available. However, since 2014 hospitals are obliged to publish standardized mortality ratios on their websites. For future research, it would be interesting to examine the relationship between mortality rates and competition within the Dutch hospital market.

## Footnotes

1 As a proxy, many papers use the number of hospitals in a given geographic radius.

2 Unfortunately, we do not have quality information of the ITCs.

3 This paragraph is partially based on [18].

4 Mandatory deductibles were €150 in 2008, €155 in 2009, €165 in 2010, and €170 in 2011. Since prices for most hospital treatments are higher than the deductible and insurers do not differentiate deductibles on the basis of the hospital chosen, patients are not price-sensitive with respect to hospital treatment.

5 Since 2013 the project organization for the Dutch Health Care Transparency Program has been integrated into the Dutch Quality Institute.

6 For a careful assessment of the second eye, there should be enough time between the surgery of the first eye and second eye.

7 Model 4 has 5 control variables, because the academic hospital dummy does not variate over the years.

8 For each year, we classified all hospitals into four quartiles, based on the number of patients treated. For cataract and bladder tumor, there was only one hospital that increased its volume so much that it moved more than two quartiles.

9 Furthermore, a simple correlation analysis shows that the simulated market share used in the IV are correlated with the original market share. In each year, the correlation coefficient is between 0.67 and 0.71 for bladder tumor, between 0.72 and 0.90 for adenoid and tonsils, and between 0.69 and 0.77 for cataract.

10 Only in the random-effects model for bladder tumor we find a significant (positive) result. The results are available from the authors on request.
